# Patient and Clinician Perspectives on the Communication of Genomic Results in Cancer Care

**DOI:** 10.1002/cam4.71287

**Published:** 2025-10-09

**Authors:** Eleanor Johnston, Zoulikha Zair, Leanna Goodwin, Louise Carter, Fiona Thistlethwaite, Matthew G. Krebs, Donna M. Graham, Kate Duffus, Emma Darlington, Natalie Cook

**Affiliations:** ^1^ The Christie NHS Foundation Trust Manchester UK; ^2^ Division of Cancer Sciences, Faculty of Biology Medicine and Health, School of Medical Sciences The Christie NHS Foundation Trust, University of Manchester Manchester UK

**Keywords:** communication, comprehensive genomic profiling, oncology, patient‐clinician communication

## Abstract

**Purpose:**

Patients diagnosed with advanced cancer are increasingly being offered comprehensive genomic profiling (CGP) to determine whether they are eligible for biomarker‐informed treatment. The communication of CGP results to patients can be suboptimal and associated with patient anxiety. This study explores patient, clinician and public experiences of CGP and preferred methods of communicating results.

**Methods:**

Focus groups were held with patients and carers, with the resulting data evaluated by thematic analysis. Concurrently, a questionnaire was designed and distributed to 60 clinicians involved in CGP studies.

**Results:**

Twenty‐four patients with a current/previous cancer diagnosis and 10 carers attended the focus groups. Experience with CGP was minimal and often limited to what participants had read on the internet. Patients/carers felt the delivery of results was very complicated and emphasised emotional facets to communicating CGP results and the wish for delivery to be tailored to them. Questionnaire responses were received from 10 UK sites. 92% of clinicians ensured patients received their CGP results, with the majority (57%) returning all CGP results, but 30% would only report on actionable mutations. Results were delivered face to face by 38% of clinicians, while other methods included letters, phone calls, or a combination of approaches. Many clinicians expressed an interest in receiving training on how to feedback CGP results.

**Conclusion:**

There is a need to develop and implement a standardised approach to returning CGP results, as well as increasing healthcare professional education and confidence with interpreting CGP. Due to the increasing access to CGP as part of routine healthcare, it is essential clinicians feel confident to interpret this information and that patients have results returned to them in an understandable format.

## 1 Introduction

In an era of precision medicine, comprehensive genomic profiling (CGP) is increasingly used in the management of cancer. CGP refers to methods used to detect genomic alterations, including somatic mutations and germline variants in cancer. The latter is most useful in testing for cancer predisposition genes in inheritable tumour types [1]. CGP is often used in the early‐phase cancer clinical trial (EPCCT) setting to ‘match’ patients diagnosed with advanced cancer to trials of novel targeted therapies. Historically, EPCCTs are designed to test the safety and tolerability of new drugs, and assess early signs of efficacy [2]; however, research has shown that biomarker‐guided EPCCTs can be associated with improved outcomes [3, 4]. Outside of the EPCCT setting, delivering CGP for patients diagnosed with cancer is a key priority of the National Health Service (NHS) in England [5]. This is delivered through the NHS Genomics Medicine Service, offering genomic testing for cancer patients according to the National Genomic Test Directory [6]. It is anticipated there will be an increasing volume of genomic testing in routine practice over the coming years.

Among EPCCTs, biomarkers are often used to ‘match’ patients to the right treatments. Consequently, these trials often include a pre‐screening element, requiring patients to have CGP to find the right biomarker or genomic alteration [2, 7, 8, 9]. All genomic results have the potential to directly impact patient care, and they can be complex and challenging for both patients and healthcare professionals (HCPs) to understand. Results are often shared with HCPs, such as oncologists and general practitioners, as well as the patients. However, limited research exists on the best methods to feedback results.

Genomic test results can be fed back via clinical letters, face‐to‐face or telephone consultations or in some instances, patients receive no information at all. Feedback of results could cause distress to patients if inadequately communicated and increase confusion about what the results mean. This is further hampered by a lack of guidance for feedback of genomic results for patients and limited evidence on patients' preferences regarding this feedback. In this study, we explored clinician, public, patients and carer perspectives on communicating genomic results.

Some research has explored HCP‐patient communication in this new era of precision oncology, but UK‐based studies exploring patient and clinician preferences regarding the feedback of CGP results are scarce. To our knowledge, this is the first exploration of clinician and patient preferences in a UK EPCCT setting.

## 2 Methods

### 2.1 Participants and Setting

We utilised a mixed methods approach to explore experiences of feedback of genomic testing results from both patient and clinician perspectives. Focus groups were held with patients diagnosed with cancer, and their relatives and carers, while a questionnaire was sent to clinicians. Both components of this project took place concurrently between March and August 2023.

This study was approved by the Christie NHS Foundation Trust local Quality Improvement and Clinical Audit Committee on 04/11/2023 (reference: 3420). We did not collect specific clinical data on a per‐patient level and therefore no additional ethical or regulatory approvals were required.

The participant population consisted of both HCPs and patients and members of the public. For the focus groups, eligible participants included those with a current or previous diagnosis of cancer, along with relatives and carers. We chose not to use strict eligibility criteria or include/exclude participants that had not previously undergone genomic testing. Participants were identified from lists of patients being treated by the EPCCT team at The Christie NHS Foundation Trust and an email distribution list for those who had signed up to be involved with projects at the cancer centre. This comprised patients, ex‐patients, family or friends of patients or individuals interested in health care. Verbal informed consent was obtained for all focus group participants. Groups were held as either in‐person sessions or over Microsoft Teams to accommodate participant preferences and availability.

The questionnaire was distributed to a mailing list of 60 clinicians across 14 NHS trusts in the UK. This included oncology consultants, nurse clinicians and trainees involved with EPCCTs on national precision medicine studies TARGET National (NCT04723316) [10] and CUPCOMP (NCT04750109) [11]. The survey was conducted on the Microsoft Forms platform [12].

### 2.2 Data Collection

The primary objective of the focus groups was to explore participant preferences regarding what types of genomic information they want to receive from clinicians, and how this should be delivered. As part of this, we evaluated existing methods of reporting results. We presented two different types of documents that could be used to communicate genomic testing results to patients. These were four pages selected from the FoundationOneCDx sample report (Appendix S1). Rather than showing the whole report, we chose specific pages as a summary of the type of information provided in the document. In presenting this, we clarified that the report would not be routinely given to patients receiving CGP. The second document was a template of a current feedback letter provided to patients as part of the ongoing TARGET National clinical trial (Appendix S2).

Focus groups opened with a question to elicit participants' preferences regarding communication with their treating medical team. Then, a short extract of a publicly available video introducing the concept of cancer genomics was played to the group [13]. Current levels of understanding, as well as interest in this topic, were discussed. Topics for the focus groups were selected based on the objectives of the project and the experiences of the group facilitators, though open discussion was encouraged by structuring the focus groups around broad themes, with prompt questions available to the group facilitators to direct discussion if needed. All focus groups were audio recorded with permission and transcribed verbatim. Each group lasted for up to 1 h.

The focus group guide can be found in Table 1.

**TABLE 1:** Focus group guide.

Focus group question guide
Introductions and housekeeping
Understanding the project, its aims and introduction to genomic testing
Patient Communication with Clinicians. How do you like to get information related to your health and your treatment? For example: Written information, emails, in‐person discussion, from online sources for example, specialist websites?
We each have our preferences on how we're given this type of information, but what is it about each of these methods that make them our preference?
Introduction to Genomics. [PLAY VIDEO UP TO 1 MINUTE 36 SECONDS: Cancer genomics | Genomics England]
Have any of you come across any of this type of information before?
Is this something that you would be interested in learning more about for yourself and your cancer?
Genomics report and patient letter review Prompts: Do you have any initial thoughts on the information that you've just looked at? How would you feel if this was related to your treatment and was given to you by your care team? How would you want to be given this type of information? ○ Is sending this via post appropriate? ○ Would you like a phone call with a clinician to talk this through? ○ Would discussing this in‐person with a clinician be appealing to you?
Would you like to be offered any extra support beyond your team to help you to understand this sort of information? Prompts: Would you like to be given information on extra resources that are available to help you learn more about genomic testing? Such as websites, or organisations that can offer support. Would you even want to go beyond the team looking after you for help understanding this type of information?

We developed and distributed a questionnaire to clinicians to understand how clinical teams feedback CGP results to their patients and how much information is given. Clinicians were also presented with seven statements related to barriers to feedback and asked to rate them on a 5‐point Likert scale.

Questionnaire items were informed by potential barriers to clinicians feeding back genomic results to their patients which were identified in existing literature [14, 15]. However, there is limited previous research in this area, and so we chose some of the statements based on the clinical practice and experience of the health care professionals. The questionnaire was anonymous but collected some demographic information including role and location. The full questionnaire with exact wording and response options is shown in Table 2.

**TABLE 2:** Questionnaire for clinicians.

Question	Response options
What is your role?	Free text
Where do you work?	Free text
Do you see/treat patients who receive genomic testing?	Yes or no
Do you always make sure all patients who receive genomic testing have their results fed back?	Yes or no
When genomic results are fed back, how much information would be given?	All results, only actionable alterations or other (free text)
When genomic results are fed back, how would you do this?	Face‐to‐face, letter/standardised letter, phone call or other (free text)
‘I don't have the capacity to keep track of all of my patients who receive testing’	5‐point Likert scale
‘There isn't enough time to feedback results to every single patient who receives testing’	5‐point Likert scale
‘I am not confident enough in my understanding of the genomic information to feed this back’	5‐point Likert scale
‘I would like to receive training on how to feedback genomic results before doing so’	5‐point Likert scale
‘I have had a bad experience feeding back genomic results’	5‐point Likert scale
‘I don't think it is important to let patients know of genomic results if there are no actionable mutations/available trials’	5‐point Likert scale
‘I am worried about the patient's reaction when genomic results are returned’	5‐point Likert scale
Have you ever had feedback from patients about their return of results?	Free text
Do you have any suggestions for improving the process of feeding back genomic results to patients?	Free text
Is there anything else you would like to mention?	Free text

*Note:* 5‐point Likert scale response options: Strongly disagree, somewhat disagree, neither agree nor disagree, somewhat agree, strongly agree.

### 2.3 Data Analysis

For analysis of the focus group results, all transcripts were first copied into a Microsoft Excel spreadsheet. Data were analysed using thematic analysis [16]. We used a ‘practical thematic analysis’ approach, as presented by Saunders et al. based on Braun and Clarke's six‐phase approach to reflexive thematic analysis. Their approach consists of three steps: (1) reading; (2) coding; and (3) theming.

In step 1, two researchers (LG and EJ), independently reviewed all transcripts and created memos. In step 2, LG and EJ and ZZ generated a list of codes and assigned each line of data appropriate codes. This iterative process involved independent reviews and code revisions. Once all data was coded, themes were drafted according to the research questions. LG, EJ and ZZ individually drafted themes and then reached a consensus.

For the questionnaire results, descriptive statistics (number and percent) were reported.

## 3 Results

### 3.1 Demographics

Seven focus groups involving 34 participants (2–8 per group) were held between April and August 2023. Twenty‐four participants were patients with a current/previous cancer diagnosis, seven were family members and three were carers. No further demographic information was collected.

Data from 37 clinicians were collated (response rate 62%). The majority of respondents were consultants (60%), followed by junior doctors (35%) and nurse consultants (5%). Responses were received from 10 of the 14 UK sites approached.

### 3.2 Focus Groups Results

Seven major themes emerged from our analysis of the focus group transcripts. Each theme and its corresponding codes are listed in Table 3.

**TABLE 3:** Themes and codes.

Themes and codes		Supporting quotes from focus groups
**1. There is a wide range of levels of familiarity with information on genomics**		‘My view about any kind of testing is that anything that can help you and anything that might help you to live longer, potentially be a cure, I'm all for anything, I will try absolutely anything because I've known from day 1 that I was incurable.’
	*1.1 Heard about genomics from the internet or own reading*	
	*1.2 Interest in learning more about genomics*	
	*1.3 Lack of understanding of genomic terminology*	
	*1.4 Participant is unfamiliar with the idea of genomics*	
	*1.5 Personal experience with genomic testing for cancer*	
**2. Some participants think that the delivery method should involve a consultation alongside providing a hard copy of the results**.		‘I'd like to have in person discussions to set the scene of what it is I'm dealing with and then it would be useful to be guided towards further information that I could go away and read… In an ideal world, maybe the chance to follow up with an e‐mail to further, you know, to ask further questions might also be really useful.’
	*2.1 Participant would like a face‐to‐face consultation, followed up with a letter/email*	
	*2.2 Participant would like a letter first, followed up by a face‐to‐face consultation*	‘I always feel I need time to assimilate that information before I have a meeting because I get a bit nervous and forget what I want to say. So, if I've read a previous report, I've got that in my head or I've got it in front of me as well that I can refer to it.’
**3. Patients view current methods of communicating information on genomic testing as inadequate**		‘Not everybody is going to want to read a 23‐page report but there is something between one line that says you have this mutation and a 23‐page report describing what it is… almost like something that gives you a summary underneath it, If you almost had an easy‐read version that said this is what this means and this is where you can find out more information’
	*3.1 I do not understand this/the document alone is not useful to me*	
	*3.2 Patients need a point of contact who is knowledgeable in genomics*	
**4. There are aspects of existing feedback mechanisms that patients like**		‘I think that is excellent, …Different trials and where they are and what they do… that is easily understandable and that would have been a real godsend in saving us a lot of worry and anxiety before we did actually get to know about relevant trials.’
	*4.1 Aspects of the current report are desirable to patients*	
	*4.2 Current letter is helpful*	
	*4.3 The current feedback letter is generic and self‐explanatory*	
**5. Delivery of genomic results should be tailored based on the patient's preferences**		‘When you think about it, the genetic mutations are moving away from this kind of one treatment approach, hopefully it will get us to personalised treatment for your specific cancer and mutation. So do the personalised communication alongside that.’
	*5.1 Communication method for genomic results should be tailored based on individual preference*	
	*5.2 Tailoring communication based on the outcome of the genomic testing*	
**6. The preferred communication method can be driven by emotion**		‘I'd also be really concerned as well to know what implications it would have for other family members’ and ‘if you do pick up something like that, that could affect other family members, not just the patient… I certainly would want that wider information’
	*6.1 An existing connection to their HCP can have an impact on patient emotions*	
	*6.2 Genomic results would make me worried about implications on family members*	
	*6.3 Patients find that the communication method has an emotional impact on the patient*	
	*6.4 The way genomic information is presented on paper can have an emotional impact on the recipient*	
	*6.5 There are mental health implications to returning genomic results*	
	*6.6 Using online resources for information related to health can have an emotional impact*	
**7. Healthcare professionals should be signposting patients to support and resources on genomics to aid understanding**		‘I think it would be useful because I think there's a great urge to go online anyway. So, if you were guiding us into ones that were going to give us accurate information I think that would be useful.’
	*7.1 Wish to be signposted to trusted resources for genomics*	
	*7.2 Interest in using trusted resources related to health information*	
	*7.3 Participants find support groups related to their cancer to be beneficial*	

*Note:* Themes in bold. Codes in italics.

### 3.3 Patient Understanding of Genomic Profiling

All codes related to participants' existing understanding of genomic testing fell under one theme: ‘**There is a wide range of levels of familiarity with information on genomics**’. Many were unfamiliar with CGP, both generally and in relation to their own diagnosis. Participants commonly queried the difference between genomics and genetics and often lacked understanding of genomics terms such as ‘mutations’. In those who were familiar with genomics, this was usually from information on the internet. Some participants had personal experience with genomic testing, such as having a mutation in the BRCA2 gene, a gene that is often detected as abnormal in hereditary breast and ovarian cancers. In another instance, a participant had enquired of their clinician if they had a certain mutation but found their clinician was unsure and unable to answer the question.

In most groups, participants expressed enthusiasm for learning more about genomics, seeing it as another ‘tool in their armour’. Subsequent results, and the discussion, will explore this in more detail.

### 3.4 Participant Review of Genomic Results Delivery Methods

When discussing communication methods, participants clearly preferred face‐to‐face consultations for ease of asking questions and building relationships with their treating team.

Some were comfortable with calls or emails for simple or nonurgent queries such as appointment times but found it hard to write down key information while on the phone. Some participants felt having a follow‐up letter was helpful for recording important information discussed during appointments that they could forget or were unable to absorb at the time. Others preferred receiving written information in advance of a consultation to prepare questions. This approach could help manage anxiety, allowing them to assimilate information beforehand.

Another theme was ‘**Patients view current methods of communicating information on genomic testing as inadequate**’. Participants expressed confusion about the meaning of the information in the documents. Many found it to be overwhelming and felt that a letter without additional information or a consultation with a qualified professional would be unhelpful. It was emphasised that information given should be as succinct as possible, with many participants commenting that they would just ‘skim read’ any letters and fixate on specific words, search them on the internet and receive incorrect information taken out of context, increasing anxiety. Participants placed a lot of value on the information being given to them in layman's terms. Some thought the material needed a summary of the main points or a link to a simplified version, for example, in the form of a diagram, video or podcast. Patients also desired a named point of contact with expertise in genomics to explain results in a way that is relatable.

Theme 4, ‘**there are aspects of existing feedback mechanisms that patients like**’ comprises comments on features of the existing documents that the focus group attendees spoke positively of. In two groups, participants found the list of clinical trials presented in the FoundationOneCDx report (Appendix S1) particularly useful.

It was almost unanimously agreed that, ideally, HCPs should personalise the delivery of genomic results to each patient, noting different levels of understanding, preferences and digital literacy skills.

### 3.5 Emotional Aspects of Returning Genomic Results

Focus group participants emphasised that communication methods have an emotive component.

One code, ‘patients find that the communication method has an emotional impact on the patient’ discusses how the format of communication used by the HCP, such as face‐to‐face or a phone call, can influence patient emotions. For instance, anxiety can arise from waiting for letters or calls. Additionally, ‘an existing connection to their HCP can have an impact on patient emotions’ explores how relationships with their treating team create trust and comfort for the patient.

Another code, ‘there are mental health implications to returning genomic results’ centred around anxiety upon receiving genomic results. The mental health impact of receiving results may vary depending on where the patient is in their treatment journey, especially if genomic results could influence patient prognosis depending on if targeted treatments are identified. Many expressed concerns about how genomic results could affect their family members and would want to know of any results that may impact their relative's future health.

### 3.6 The Need for New Trusted Educational Resources

Many participants felt that they would benefit from a resource that could ‘decode’ complex genomic information.

### 3.7 Responses to the Clinician Questionnaire

#### 3.7.1 How Clinicians Currently Deliver Genomic Testing Results in Their Practice

Appendix S3 outlines all responses to the clinician questionnaire.

All respondents had patients under their care who receive CGP, and the majority (92%) responded stating that they ensure their patients received feedback on genomic results. When feeding back results to their patients, 57% of clinicians would feedback all genomic results, whilst 30% would only report on actionable mutations. The remaining respondents, answering in free text (13%), primarily mentioned feeding back actionable results as well as any relevant germline mutations that may require follow up. One respondent commented that many will not have actionable results, so they would state which actionable results are absent.

With regard to the methods used by clinicians to feed back results to their patients, the majority (38%) delivered results face‐to‐face, 16% delivered results via a letter and 14% via phone call. The remaining 32% used a combination of methods, with deciding factors including the results themselves and clinical circumstances.

#### 3.7.2 Clinician‐Perceived Barriers to Returning Genomic Results

**FIGURE 1:**
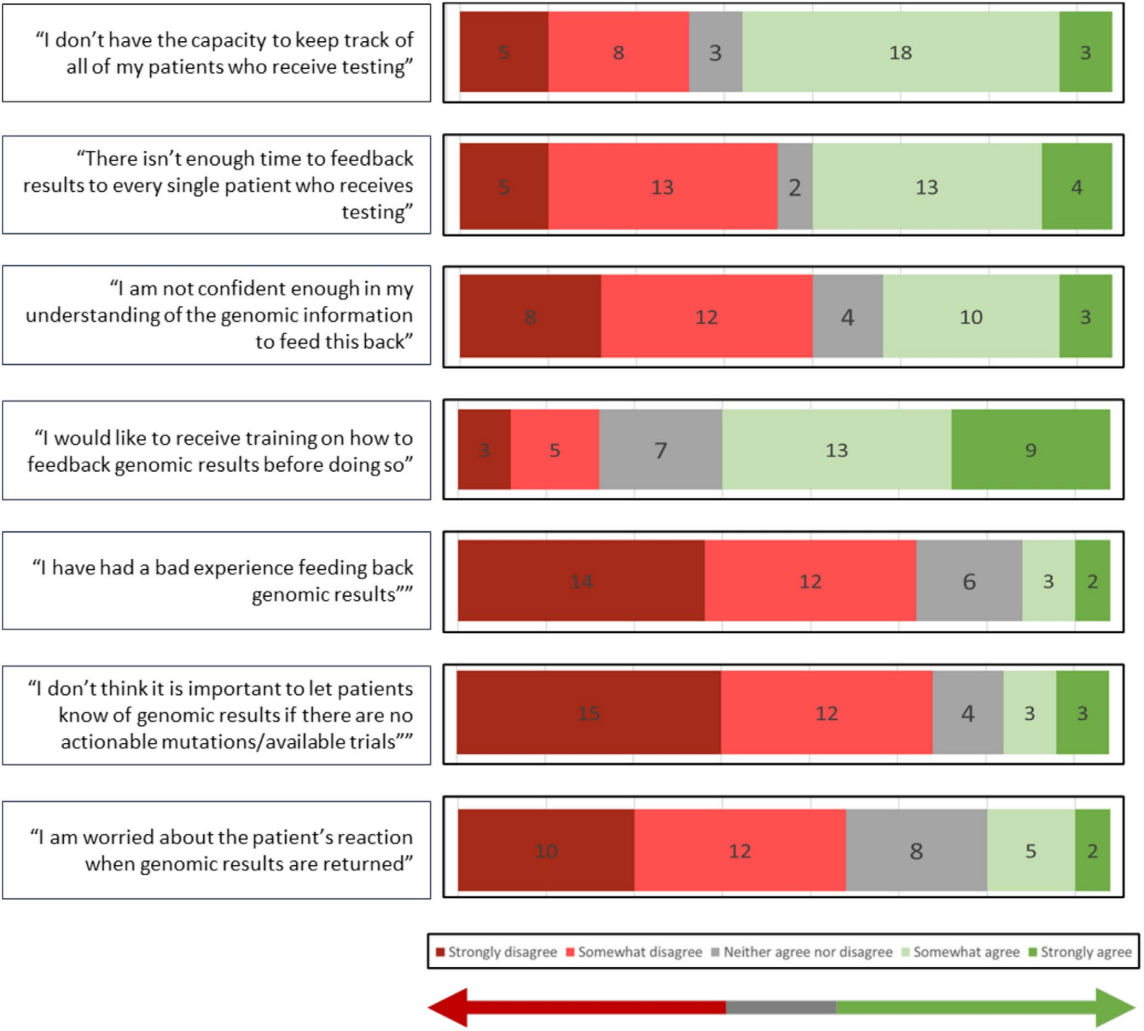
Responses to the statements addressing potential barriers to clinician feedback of genomic results. Respondents were asked to ‘rate’ each statement between strongly disagree and strongly agree.

Potential barriers to the feedback of CGP results were explored within the questionnaire. The responses to all seven statements are demonstrated in Figure 1.

#### 3.7.3 Clinician Suggestions for Process Improvement

With regards to improving the process of delivering CGP results, several clinicians suggested education and training for medical staff, and one mentioned the potential value of a crib sheet for nurses to help explain results or answer questions after their patients have been given results from the doctors. Other suggestions included the use of additional material to accompany the return of results, such as a video or leaflet. This resource could be used to explain genomic results in more detail, and in language patients would understand.

A significant proportion of participants expressed interest in receiving training on how to effectively communicate genomic results, though there was some variation in confidence levels regarding the understanding of genomic information, with many respondents indicating low confidence in their understanding of genomic information (Table 4).

**TABLE 4:** Summary of key results from clinicians, patient and public perspective.

Summary of key results from clinician, patient and public perspective	
Clinician	Patient/Public
Lack of understanding of different types of genomic changes, and how testing results can affect their treatment options.	Large amount of variability in understanding of genomics in the context of cancer care
Training in understanding genomic results and how better to communicate genomic results	Patients are often reliant on HCPs to deliver information in a way that is meaningful to them
Having a standardised letter and/or other resources such as a leaflet or video would help HCPs in their communication and support patient understanding	The language used in the delivery of genomics results, in whatever format, should be simplified as much as possible
Patient sometimes misunderstand of what the test is for and what it shows, there is a need to manage patient expectations prior to performing the CGP	Lack of understanding of different types of genomic changes, and how testing results can their affect treatment options
Access to MTBs is valued to discuss the significance of different results	Value being directed to reliable and trusted websites, videos and information leaflets
Value in implementation of genomic data managers/a dedicated person to manage the workload	Value in a ‘named contact’ as a reference point for specific questions related to genomics
Important not to duplicate or add to the burden of HCP work—feedback of results can be very time consuming	Communication that is tailored to their preferences and needs
	Anxiety around genomic results and impact on care, as well as the impact on relatives

## 4 Discussion

In this study, we sought to understand communication methods when returning genomic results, aiming to recommend an approach for feedback of results that can be incorporated into standard practice. Improving patient understanding of genomic testing is crucial to ensure patients fully comprehend test results and are empowered to make informed decisions about their care.

Exploring the priorities and needs of patients and their HCPs continues to grow in importance as the landscape of the molecular and genomic profiling of cancer continues to evolve. Different types of testing are changing the way we understand and treat cancer, and we are now able to profile complex molecular characteristics of individual tumours, including but not limited to CGP, GEP and protein expression patterns [8, 17]. This has revolutionised the treatment of some types of cancers; however, there has been limited research investigating the understanding and communication of these results by healthcare teams [18, 19].

Two previous reviews have explored evidence around patient and oncologist knowledge of, confidence in and communication preferences surrounding complex testing in precision oncology [14, 20]. The first explored the literature on patient and oncologists' knowledge and attitudes regarding tumour multigene next‐generation sequencing (NGS). The second summarised evidence regarding comprehensive biomarker testing‐related communication between people impacted by cancer and HCPs. Both reviews found that although patients had generally poor subject knowledge, they were often interested in biomarker testing/NGS, though patient expectations about having actionable findings and receiving access to targeted treatments on the basis of test results were often higher than is realistic. Outside of a cancer setting, other prior studies have explored the return of genomic results in multiple disease types, including non‐malignant incurable disease [21, 22, 23].

In the US, other recent research has explored patient experiences with biomarker testing in patients with lung, breast and colorectal cancer [24] and non‐small cell lung cancer [25]. Similar to our findings, both of these studies concluded that educational material would be valuable to improve patient knowledge.

A UK‐based study by Tutika et al. utilised a survey to explore the current understanding of cancer genomics among 150 UK oncologists [15]. They identified there was a lack of training among oncologists, and only a third of respondents had good knowledge of how to explain genomic concepts to patients. There was a clear need for further specific training in cancer genomics. Subsequent to this survey, NHS England created GeNotes, a training resource created in collaboration with clinicians, though this is not specific to cancer [26].

There is also scarce literature exploring patients' understanding and attitudes towards genomic testing across cancer types in an early phase trials setting. Fallowfield et al. recently explored how 2 short videos could be useful in improving patient knowledge and aid decision making towards gene expression profiling (GEP) testing in patients with early‐stage oestrogen receptor positive (ER+) breast cancer [27]. Through their phase four clinical trial, they found the videos significantly increased knowledge and understanding of GEP tests compared to those receiving the standard industry‐provided information leaflets, showing a role for educational videos in this population. It would be useful to explore this in the EPCCT setting.

Our focus groups revealed variation in public familiarity with genomics, with many participants unfamiliar with its relevance to cancer treatment. Additionally, our clinician questionnaire indicated a gap in HCPs' understanding and an interest in training in genomic testing in cancer. This was reinforced by the focus groups' results, which revealed patients' expectations for their treating team to explain results in relatable terms and answer any questions. Many focus group participants wanted to learn more about genomic testing in the context of their own cancer care, acknowledging its potential benefits. However, the necessity of all language used in communication being accessible to a lay person was emphasised; crucially, participants valued clarity about the implications of results.

Ultimately, many focus group participants felt that delivering patients' results should be tailored to individual preferences. However, we must acknowledge that this may not always be feasible. Growing public healthcare pressures and the increasing uptake of testing within the NHS Genomics system make it impractical for clinicians to deliver face‐to‐face consultations for every patient. A priority must be ensuring CGP results are contextualised for patients and facilitating their understanding of a summary letter. Providing this for every individual would be complex and time‐consuming for HCPs, impacting clinical service. One suggestion for delivery includes introducing specialist nurse‐led clinics for returning genomics results to patients, as has been done in oncology, though further education and training would still be required for HCPs involved. It is also important to consider that any new processes should not increase the burden on patients who undergo CGP as part of their care; any changes should reduce time toxicity to patients.

The delivery of any healthcare‐related information often has major implications for the patient, particularly regarding prognosis. In an EPCCT setting, many patients receiving testing have exhausted standard treatment options and may hope for a novel ‘matched’ treatment approach. So, it is critical that clinicians can manage patient expectations. This issue was raised by one of the clinicians in the questionnaire, noting that ‘expectations of testing resulting in a suitable treatment are high and often unrealistic.’ This point, as well as others raised within the clinician questionnaire, relates not only to results delivery but also to the consent process, as the improvement of patient understanding of genomic testing should occur at the point of informed consent.

This research was conducted at a single centre, and although the clinician questionnaire included responses from across the country, we did not receive responses from all the sites or recipients. While the majority of clinician respondents claimed to ensure that patients received their genomic results, we must acknowledge the self‐selecting nature of the question, as it is impossible to know if those who did not respond had less experience or did not deliver results to patients.

Other limitations include a small sample size, a lack of information from focus groups on baseline education, experience of CGP and other factors influencing genomic literacy. The same facilitators were present for all focus groups to ensure consistency, however as they were not independent from the project team, we are aware this could contribute to a degree of unconscious bias. Additionally, although we recognise best practice is to conduct thematic analysis until data saturation is reached, we found that by the final groups much of the discussion was repeating insights and patterns from the prior groups. We also acknowledge our focus group participants were not representative of the Greater Manchester population, and further work is ongoing to address this and bring more diverse voices into the ongoing work. Despite these limitations, to our knowledge this is the first piece of work exploring this subject in the UK and our findings provide valuable insight into the perspectives of clinicians and patients, providing a solid foundation for future work.

The findings of this research have the potential to influence clinical practice. It is clear that we need to optimise the delivery of CGP results to patients and upskill HCPs involved in feedback, alongside educating patients and the public. New processes could be embedded into national precision medicine initiatives, which provide targeted and/or whole genome sequencing approaches across the UK. Furthermore, this work can inform how complex results are shared with patients in general as we have gauged patient priorities. Moving forward, we aim to create a patient‐accessible resource in the form of a short, easy‐to‐understand animation with an accompanying leaflet explaining CGP in the cancer setting, detailing how patients are matched to treatments or clinical trials. These will be created in collaboration with patient and public representatives and made freely available and accessible, including through trusted websites, such as the NHS Genomics Service and trusted charities. The aim is that these resources will guide and support helpful conversations between HCPs and those considering CGP or receiving results as part of their cancer care pathway.

## Author Contributions

**Eleanor Johnston:** writing – original draft, project administration, formal analysis, writing – review and editing, investigation, methodology, data curation. **Zoulikha Zair:** writing – review and editing, formal analysis, investigation. **Leanna Goodwin:** writing – review and editing, formal analysis, investigation, methodology. **Louise Carter:** writing – review and editing. **Fiona Thistlethwaite:** writing – review and editing. **Matthew G. Krebs:** writing – review and editing. **Donna M. Graham:** writing – review and editing. **Kate Duffus:** writing – review and editing. **Emma Darlington:** writing – review and editing, funding acquisition. **Natalie Cook:** conceptualization, supervision, methodology, writing – review and editing, formal analysis, funding acquisition, investigation.

## Ethics Statement

Ethics approval for this study was not required as we did not collect specific clinical data on a per‐patient level and the project falls under the category of audit or service evaluation.

## Consent

Verbal informed consent was obtained for all focus group participants.

## Conflicts of Interest

N.C. has received consulting fees from Roche Pharmaceuticals, Servier and REDX Pharmaceuticals; payment or honoraria for lectures, presentations, speakers' bureaus, manuscript writing or educational events from Roche Pharmaceuticals; support for attending meetings and/or travel from Roche Pharmaceuticals; research funding to the research team from AstraZeneca, Orion, F. Hoffmann‐La Roche Ltd., Taiho, Novartis, Starpharma, Bayer, Eisai, UCB, RedX Pharmaceuticals, LOXO‐oncology, Avacta, Boehringer Ingelheim, Merck and Tarveda Therapeutics; participated in a data safety monitoring board or advisory board at Roche Pharmaceuticals and Cancer Research UK. L.C. has had consultancy roles for Athenex, Bicycle Therapeutics and Boehringer Ingelheim. She has received institutional funding from Sierra Oncology, Athenex, Takeda, CellCentric, CytomX Therapeutics, Eli Lilly, Boehringer Ingelheim, Bicycle Therapeutics, Lupin Pharmaceuticals, Repare Therapeutics, ADC Therapeutics, Merck Serono and Kronos Bio. M.G.K. has received honoraria from Janssen, Roche; consulting/advisory board fees from Bayer, Ellipses Pharma, Guardant Health, Janssen, OM Pharma, Roche, Seattle Genetics; speaker fees from AstraZeneca, Janssen, Roche and research funding from Roche and Novartis for his institution. M.G.K. has received travel, accommodation or expenses from AstraZeneca, BerGenBio, Immutep, Janssen and Roche. D.G. has received research funding from: AstraZeneca and institutional research funding from: MSD, Codiak Biosciences, Starpharma, Faron Pharmaceuticals, Synthon, Janssen, AstraZeneca, Roche, BerGenBio, GSK, Bayer, Bicycle Therapeutics, Carrick, Taiho Pharmaceuticals, CytomX Therapeutics, RedX Pharma PLC, Eisai, Octimet, Orion Pharma, Kinex Pharmaceuticals, Boehringer Ingelheim, BMS, Turning Point Therapeutics, Immutep, Agalimmune, Kymab Ltd./Sanofi, Blueprint, Astellas, Cellcentric, UCB, Eli Lilly, Seagen, Repare Therapeutics, Timepoint Therapeutics, Astex, Stemline, Crescendo Biologics Ltd., ADC Therapeutics, Genentech, Avacta Life Sciences Ltd., Nurix Therapeutics, Chugai Pharmaceuticals, Incyte. F.T. has research funding from: GSK, Novartis and institutional research funding from Seagen, Grey Wolf Therapeutics, GenMab, CytomX, Incyte, Janssen, Adaptimmune, BMS, Immunocore, Achilles Ltd., Oxford Vacmedix, RS Oncology LLC, Roche, Kymab Ltd/Sanofi, Chugai, T‐Knife, Novalgen, Nucana, Biontech, Moderna, Zymeworks. F.T. has personal income from advisory board/consultancy/honoraria/DSMB: Astrazeneca, BMS, GSK, T‐Knife Therapeutics, Janssen, Immatics, Ixaka, Scenic Biotech, F‐Star, Kite, Leucid and Guidepoint. The remaining authors declare no conflicts of interest.

## Supporting information


**Appendix S1:** cam471287‐sup‐0001‐AppendixS1.docx.

**Appendix S2:** cam471287‐sup‐0002‐AppendixS2.docx.

**Appendix S3:** cam471287‐sup‐0003‐AppendixS3.docx.

## Data Availability

Full transcripts can be shared upon request. Patient details would not be available.

## References

[1] W. S. El‐Deiry , R. M. Goldberg , H. J. Lenz , et al., “The Current State of Molecular Testing in the Treatment of Patients With Solid Tumors, 2019,” CA: A Cancer Journal for Clinicians 69, no. 4 (2019): 305–343, 10.3322/caac.21560. 31116423 PMC6767457

[2] E. Fountzilas , A. M. Tsimberidou , H. H. Vo , and R. Kurzrock , “Clinical Trial Design in the Era of Precision Medicine,” Genome Medicine 14, no. 1 (2022): 101, 10.1186/s13073-022-01102-1. 36045401 PMC9428375

[3] M. Schwaederle , M. Zhao , J. J. Lee , et al., “Association of Biomarker‐Based Treatment Strategies With Response Rates and Progression‐Free Survival in Refractory Malignant Neoplasms: A Meta‐Analysis,” JAMA Oncology 2, no. 11 (2016): 1452–1459, 10.1001/jamaoncol.2016.2129. 27273579

[4] M. Schwaederle , M. Zhao , J. J. Lee , et al., “Impact of Precision Medicine in Diverse Cancers: A Meta‐Analysis of Phase II Clinical Trials,” Journal of Clinical Oncology 33, no. 32 (2015): 3817–3825, 10.1200/JCO.2015.61.5997. 26304871 PMC4737863

[5] NHS , “NHS Long Term Plan,” (2019), https://www.longtermplan.nhs.uk/online‐version/chapter‐3‐further‐progress‐on‐care‐quality‐and‐outcomes/better‐care‐for‐major‐health‐conditions/cancer/.

[6] NHS England , “The National Genomic Test Directory,” (2024), https://www.england.nhs.uk/publication/national‐genomic‐test‐directories/.

[7] J. Mateo , L. Steuten , P. Aftimos , et al., “Delivering Precision Oncology to Patients With Cancer,” Nature Medicine 28, no. 4 (2022): 658–665, 10.1038/s41591-022-01717-2. 35440717

[8] E. R. Malone , M. Oliva , P. J. B. Sabatini , T. L. Stockley , and L. L. Siu , “Molecular Profiling for Precision Cancer Therapies,” Genome Medicine 12, no. 1 (2020): 8, 10.1186/s13073-019-0703-1. 31937368 PMC6961404

[9] T. H. Carr , R. McEwen , B. Dougherty , et al., “Defining Actionable Mutations for Oncology Therapeutic Development,” Nature Reviews Cancer 16, no. 5 (2016): 319–329, 10.1038/nrc.2016.35. 27112209

[10] “Tumour Characterisation to Guide Experimental Targeted Therapy—National,” (2024), clinicaltrials.gov https://clinicaltrials.gov/study/NCT04723316.

[11] “Carcinoma of Unknown Primary (CUP): A Comparison Across Tissue and Liquid Biomarkers (CUP‐COMP),” (2022), clinicaltrials.gov https://clinicaltrials.gov/study/NCT04750109.

[12] Microsoft , “Microsoft Forms,” https://forms.microsoft.com/.

[13] Genomics England , “Understanding Cancer Genomics (v. 1.0 15/05/2017),” (2017), https://www.youtube.com/watch?v=au1EBIpk6ec.

[14] M. Shirdarreh , O. Aziza , R. C. Pezo , K. J. Jerzak , and E. Warner , “Patients' and Oncologists' Knowledge and Expectations Regarding Tumor Multigene Next‐Generation Sequencing: A Narrative Review,” Oncologist 26, no. 8 (2021): e1359–e1371, 10.1002/onco.13783. 33823080 PMC8342587

[15] R. K. Tutika , J. A. Bennett , J. Abraham , et al., “Mainstreaming of Genomics in Oncology: A Nationwide Survey of the Genomics Training Needs of UK Oncologists,” Clinical Medicine 23, no. 1 (2023): 9–15, 10.7861/clinmed.2022-0372. 36697012 PMC11046524

[16] C. H. Saunders , A. Sierpe , C. von Plessen , et al., “Practical Thematic Analysis: A Guide for Multidisciplinary Health Services Research Teams Engaging in Qualitative Analysis,” BMJ 381 (2023): e074256, 10.1136/bmj-2022-074256. 37290778

[17] S. L. Rulten , R. P. Grose , S. A. Gatz , J. L. Jones , and A. J. M. Cameron , “The Future of Precision Oncology,” International Journal of Molecular Sciences 24, no. 16 (2023): 12613, 10.3390/ijms241612613. 37628794 PMC10454858

[18] O. D'Oria , A. Giannini , A. R. Besharat , and D. Caserta , “Management of Endometrial Cancer: Molecular Identikit and Tailored Therapeutic Approach,” Clinical and Experimental Obstetrics and Gynecology 50, no. 10 (2023): 210.

[19] I. Cuccu , O. D'Oria , L. Sgamba , et al., “Role of Genomic and Molecular Biology in the Modulation of the Treatment of Endometrial Cancer: Narrative Review and Perspectives,” Healthcare (Basel) 11, no. 4 (2023): 571, 10.3390/healthcare11040571. 36833105 PMC9957190

[20] T. Pichler , F. Mumm , N. Dehar , et al., “Understanding Communication Between Patients and Healthcare Professionals Regarding Comprehensive Biomarker Testing in Precision Oncology: A Scoping Review,” Cancer Medicine 13, no. 3 (2024): e6913, 10.1002/cam4.6913. 38298115 PMC10905543

[21] D. F. Vears , J. T. Minion , S. J. Roberts , J. Cummings , M. Machirori , and M. J. Murtagh , “Views on Genomic Research Result Delivery Methods and Informed Consent: A Review,” Personalized Medicine 18, no. 3 (2021): 295–310, 10.2217/pme-2020-0139. 33822658 PMC8242984

[22] D. F. Vears , J. T. Minion , S. J. Roberts , et al., “Return of Individual Research Results From Genomic Research: A Systematic Review of Stakeholder Perspectives,” PLoS One 16, no. 11 (2021): e0258646, 10.1371/journal.pone.0258646. 34748551 PMC8575249

[23] S. Fontoura Dias , M. Barbosa , F. Júlio , et al., “Communicating Genetic Information in Families With Inherited Late‐Onset Neurodegenerative Diseases: A Scoping Review,” Health Communication (2025): 1–23, 10.1080/10410236.2025.2475565. 40135509

[24] E. E. Fortune , A. K. Zaleta , and M. C. Saxton , “Biomarker Testing Communication, Familiarity, and Informational Needs Among People Living With Breast, Colorectal, and Lung Cancer,” Patient Education and Counseling 112 (2023): 107720, 10.1016/j.pec.2023.107720. 37062167

[25] A. Pack , A. Russell , S. Kircher , et al., “Current Communication Practices for Biomarker Testing in Non‐Small Cell Lung Cancer: Exploring Patient and Clinician Perspectives,” Patient Education and Counseling 114 (2023): 107839, 10.1016/j.pec.2023.107839. 37321114 PMC10528088

[26] A. Frost , A. Kelly , M. Bishop , et al., “Genotes—a “Just‐In‐Time” Genomics Education Resource Co‐Designed With Clinicians,” BMC Medical Education 24, no. 1 (2024): 1378, 10.1186/s12909-024-06059-w. 39593035 PMC11600734

[27] L. Fallowfield , I. Solis‐Trapala , R. Starkings , L. Matthews , S. May , and V. Jenkins , “Improving Patient Understanding of GEP Test Results (IMPARTER4): An RCT,” BMJ Oncology 4, no. 1 (2025): e000689, 10.1136/bmjonc-2024-000689. PMC11931957 40130222

