# Supplementary figures and images for: Patient and Clinician Perspectives on the Communication of Genomic Results in Cancer Care
Source: Cancer Med. 2025 Oct 9;14(19):e71287. doi: 10.1002/cam4.71287 (PMC12509244; doi:10.1002/cam4.71287)

Appendix 1. Foundation medicine report used in the focus groups.


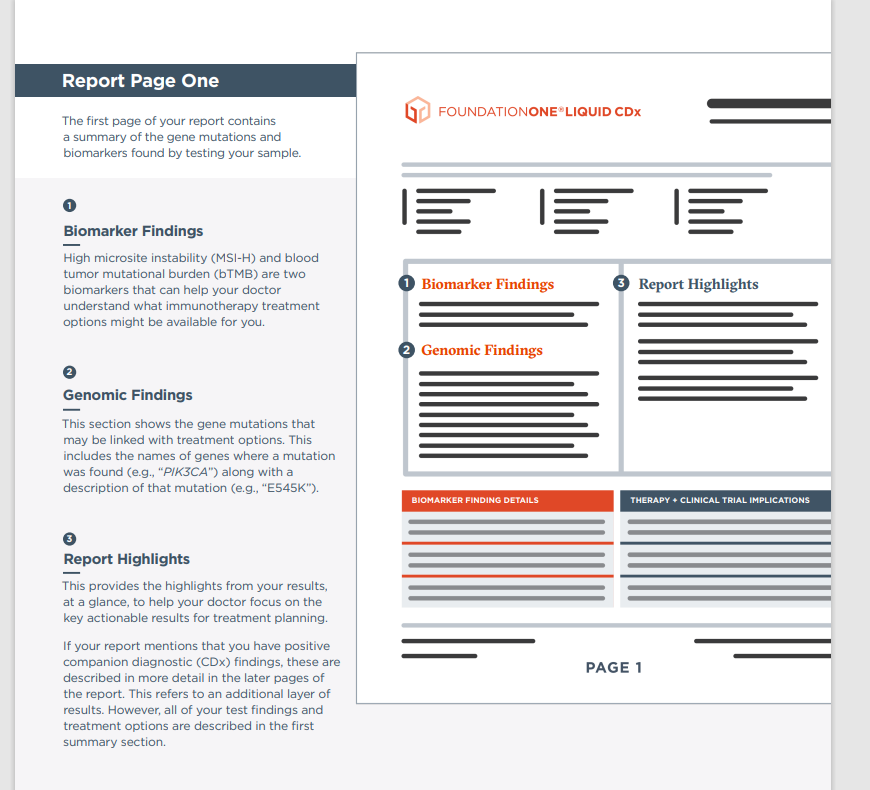


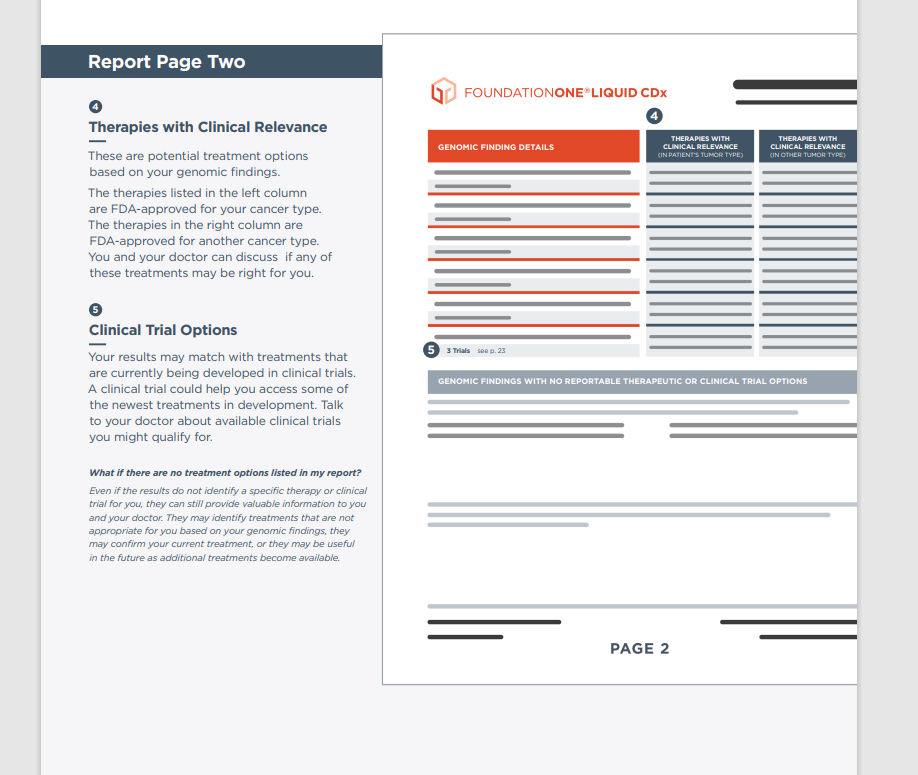

Supplement: Supplementary file 1 — Appendix S1: cam471287‐sup‐0001‐AppendixS1.docx. [file CAM4-14-e71287-s003.docx]

Appendix 2. TARGET National feedback letter


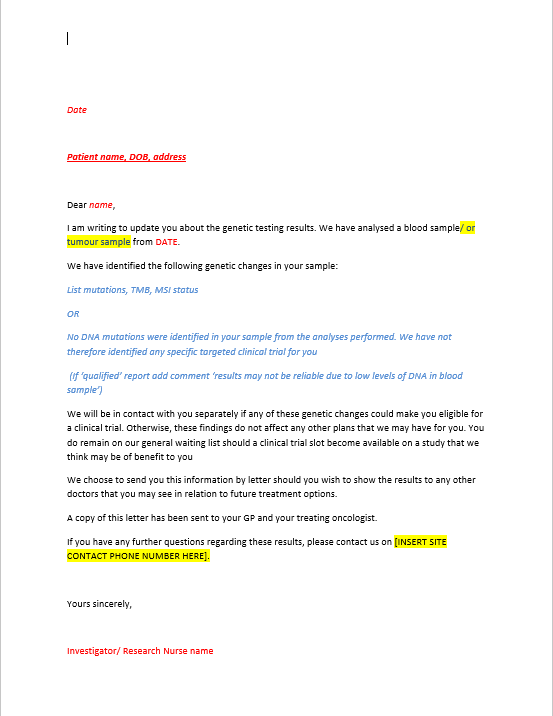

Supplement: Supplementary file 2 — Appendix S2: cam471287‐sup‐0002‐AppendixS2.docx. [file CAM4-14-e71287-s001.docx]
