# Supplementary material for: Patient and Clinician Perspectives on the Communication of Genomic Results in Cancer Care
Source: Cancer Med. 2025 Oct 9;14(19):e71287. doi: 10.1002/cam4.71287 (PMC12509244; doi:10.1002/cam4.71287)
Supplement: Supplementary file 3 — Appendix S3: cam471287‐sup‐0003‐AppendixS3.docx. [file CAM4-14-e71287-s002.docx]

Appendix 3 – full clinician survey and results

**Q1. What is your role?**

| **Response** | **Number of respondents (%)** |
| --- | --- |
| Consultant | 22 (60) |
| Junior Doctor | 13 (35) |
| Nurse Consultant | 2 (5) |
| Total | 37 |

**Q2. Where do you work?**

| **Response** | **Number of respondents (%)** |
| --- | --- |
| Bath | 1 (3) |
| Cambridge | 1 (3) |
| Cardiff | 1 (3) |
| Christie | 18 (49) |
| Devon | 1 (3) |
| Edinburgh | 2 (5) |
| Glasgow | 2 (5) |
| Leeds | 1 (3) |
| Liverpool | 2 (5) |
| Newcastle | 4 (11) |
| Unknown | 4 (11) |
| Total | 37 |

**Q3. Do you see/treat patients who receive genomic testing?**

| **Response** | **Number of respondents (%)** |
| --- | --- |
| Yes | 37 (100) |
| No | 0 |

**Q4. Do you always make sure all patients who receive genomic testing have their results fed back?**

| **Response** | **Number of respondents (%)** |
| --- | --- |
| Yes | 34 (92) |
| No | 3 (8) |
| Total | 37 |

**Q5. When genomic results are fed back, how much information would be given?**

| **Response** | **Number of respondents (%)** |
| --- | --- |
| All results | 21 (57) |
| Only actionable alterations | 11 (30) |
| Other* | 5 (13) |

**Other responses:*

1. *Actionable results mentioned, general of others, germline if relevant. Full report in notes and to main consultant.*
2. *Actionable, drivers, any requiring germline FU*
3. *And any possible germline mutations that need further testing*
4. *Many will not have actionable results, so state which actionable results are absent.*
5. *results of relevance to that individual patient, or to their family members if they wish this discussed.*

**Q6. When genomic results are fed back, how would you do this?**

| **Response** | **Number of respondents (%)** |
| --- | --- |
| Face-to-face | 14 (38) |
| Letter/standardised letter | 6 (16) |
| Phone call | 5 (14) |
| Other* | 12 (32) |

**Other responses:*

1. *Any of the above depending on the context of the consultation/results from profiling*
2. *Both face to face and virtual appointment*
3. *Call/clinic with patient. Letter with full report to home consultant*
4. *Depends on scenario. Written information always provided by letter or report but may also be face to face if patient in clinic or by telephone if there is something that needs further clarification or a potential treatment is available.*
5. *Either F2F or phone depending on patient preference and results*
6. *f2f or phone*
7. *face to face or phone call depending how far patient is from hospital.*
8. *Face to face or phone call or letter*
9. *face to face or phone call, sometimes backed up by a letter, depending on clinical circumstances.*
10. *Face to face or TC or letter*
11. *Letter or call. If coming for face to face for another reason will have a consultation, then.*
12. *mixture. I also believe that the team requesting the test should be responsible for informing the patient.*

**Q7. Series of statements**

|  | Strongly disagree | Somewhat disagree | Neither agree nor disagree | Somewhat agree | Strongly agree |
| --- | --- | --- | --- | --- | --- |
| “I don’t have the capacity to keep track of all of my patients who receive testing” | 5 | 8 | 3 | 18 | 3 |
| “There isn’t enough time to feedback results to every single patient who receives testing” | 5 | 13 | 2 | 13 | 4 |
| “I am not confident enough in my understanding of the genomic information to feed this back” | 8 | 12 | 4 | 10 | 3 |
| “I would like to receive training on how to feedback genomic results before doing so” | 3 | 5 | 7 | 13 | 9 |
| “I have had a bad experience feeding back genomic results” | 14 | 12 | 6 | 3 | 2 |
| “I don’t think it is important to let patients know of genomic results if there are no actionable mutations/available trials” | 15 | 12 | 4 | 3 | 3 |
| “I am worried about the patient’s reaction when genomic results are returned” | 10 | 12 | 8 | 5 | 2 |

**Q8. Have you ever had feedback from patients about their return of results?**

| **Response** | **Number of respondents (%)** |
| --- | --- |
| No | 22 (60) |
| Yes (no further clarification given) | 3 (8) |
| Other* | 12 (32) |

**Other responses:*

1. *Feedback has been that they have been happy to receive and those that are particularly interested generally ask for a copy of the report. Some issues around complicating line of care and the testing and results can cause some confusion about who is leading their cancer care.*
2. *Generally positive*
3. *No - patients in CUP still don't seem to understand even if it leads to different treatments/trials.*
4. *no but always check understanding and any questions.*
5. *patients are mostly appreciative, even if there are no actionable mutations.*
6. *Patients are often worried about implications on family. Patients have commented that it is not easy to understand.*
7. *The genomic testing in my field is mainly for syndromes and trials. Patients are relieved if their genetic testing is negative for familiar cancer syndromes.*
8. *The potential germline mutations cause anxiety. The variations of uncertain significance so they can't have a treatment, but you don't know it wouldn't work for them are always challenging too.*
9. *They have struggled to interpret findings over the phone only.*
10. *Yes - I would say patients are often frustrated that mutations are not actionable, or that a treatment could be available but is not on the NHS.*
11. *yes, when it has been through a pre-screening I have arranged.*
12. *Yes. Mostly positive*

**Q9. Do you have any suggestions for improving the process of feeding back genomic results to patients?**

| **Response** | **Number of respondents (%)** |
| --- | --- |
| No | 7 (19) |
| Other* | 30 (81) |

**Other responses:*

1. *A leaflet to explain in more detail would be good. It could go out with the letter.*
2. *A link or information sheet with a lay description of genomic results, what they do (and don't) mean and the caution with interpreting any associated trial suggestions in the report.*
3. *A standardised, patient / GP / non-specialist - friendly report that can be added to their electronic record and copied to the patient would be very helpful. It would need to be in lay terms, only mention areas that may have clinical relevance, and suggest discussion with their oncologist. Important not to overplay the result, as patients may now be too ill for treatment, or there may not be an approved drug or open trial that is suitable.*
4. *Ask the patients opinion. Use of videos to explain.*
5. *Availability of standardised letters*
6. *Clinician should get some training with this.*
7. *Current letter format is not user friendly in my opinion.*
8. *Establish patient expectations at the time of doing the tests.*
9. *Giving results in paper format and discussing it with them.*
10. *Having a Patient Information leaflet on what is genomic testing would be useful (like an infographic)*
11. *I don’t think information that is not relevant for the treatment (or to diagnose/exclude syndromes) should be thrown at the patient with details, could be resumed as “other alterations that don’t have a corresponding treatment”.*
12. *I often feel patients do not understand the genomic results and they often think the test it is to try to find the primary cancer (in patients with CUP)*
13. *I think patients would appreciate if the clinically relevant results were explained in a simpler way.*
14. *If patients were fed back their standard of care genomic testing in a more explicit way that would help to improve genomic literacy making feedback of more complex testing easier.*
15. *In relation to question 7, results are usually fed back to patients at the time of their clinics, so it doesn't require additional clinic time. Once results come back to clinicians, they are entered into the mutation analysis form on CWP (The Christie, Manchester) and fed back to the patient at next clinic.*
16. *Include counselling on the likelihood of inactionable mutations prior to testing.*
17. *Keep oncologists UpToDate in interpreting results as this moves forwards, I find TMB meetings invaluable to help me interpret results before I discuss with patients.*
18. *Maybe a crib sheet for nurses to help explain results or answer questions after they patients has been given results from the doctors.*
19. *Maybe a standardised letter will help.*
20. *More training for clinical staff on how to interpret results and more time allocated to discuss them, as most patient don't fully understand standardised letter and often get quite anxious.*
21. *Needs to be comprehensive.*
22. *Not particularly. It's time consuming but usually ok. I'm not sure beyond additional info what would make a difference for patients.*
23. *Only actionable and germline mutations to be fed back.*
24. *Patients should be told implications of these findings the chances of getting targeted treatment.*
25. *The formal results are very comprehensive and too much to give patients. Results being feedback perhaps should be more concise focusing on no actionable results or actionable results. A generic PIS explaining what these could mean would be useful.*
26. *To discuss results first in an MTB to be sure what the different mutations mean in terms of targets and function in case this isn't obvious from the report.*
27. *Training for medical staff would be useful as this is new to many of us and we are learning "on the job”.*
28. *Training would be useful to help with ideas appropriate terminology to use.*
29. *We need genomic data managers. the pathways are not well defined and often require chasing. Probably ought to automate incorporation into CWP.*
30. *Written information in language patients understand would be helpful to accompany the conversation - links to videos etc. I suspect education in all levels of medical staffing/nursing roles also required to ensure same information shared by all parties.*

**Q10. Is there anything else you would like to mention?**

| **Response** | **Number of respondents (%)** |
| --- | --- |
| No | 23 (62) |
| Other* | 14 (38) |

**Other responses:*

1. *As above- we need to smooth/automate the pathway.*
2. *Ethics on telling relatives about germline mutations when the patient does not want to know.*
3. *Expectations of testing resulting in a suitable treatment are high and often unrealistic.*
4. *"I feel sometimes I don't fully understand the results if they are not actionable, this is something I would like to learn more about.*
5. *I have answered "Somewhat agree" to capacity/feedback but truth is it is very time-consuming, and I am at full capacity so limit the testing I do and patient I see. It is at least 4h work per patient and I just can't find that time if not directly linked to trials we are running here. I can't follow-up on germline results etc so now have a strict system of feeding those back with advice for their main consultant to action and follow-up. I am trying to support other consultant to integrate testing access into their own practice to increase capacity, but they are intimidated by both the knowledge and time requirements. Training the next generation will clearly be key as this rapidly expands across the whole service.*
6. *I strongly recommend sharing genomics results with patients, but in simple terms and only for the driver mutations that have an impact on choosing appropriate SOC regimens in clinic. This is part of patient centred care. Patients are better informed now and most of them would wonder anyway whether these tests are being done as part of their management? it is good to reassure them if we are doing this in routine practice.*
7. *In CUP patients still think genomic testing is about finding the primary cancer*
8. *Information to patient needs to go beyond an enumeration of findings. some contexts will help. Maybe something modelled of the reports we get from St Marys which talk about the use or potential implications of some of the mutations.*
9. *It would be helpful to have a dedicated person in each disease group who could upload results to CWP and complete "Mutation analysis" form on CWP (The Christie).*
10. *Results need to be fed back in a way patients can understand. But also need to not duplicate or add to the burden of clinician work - this can be very time consuming and create a marked increase in workload which reduces the time that can be spent delivering these.*
11. *There is still significant need for reports to be uploaded to centralised medical notes where any clinical staff member can access it. Most reports are stored in inaccessible databases, or sent only by email, and this hampers the ability to access test results for downstream clinicians.*
12. *Training and educational material with access would improve clinicians understanding and confidence which will ultimately benefit patients.*
13. *Training would be an excellent idea, at least a video course.*
14. *we have a genomics navigator as a pilot post which has enabled us to be confident that all our genomic requests are managed in a timely manner and the results available in one place. This has supported being able to give results back to patients complete and in a timely manner.*
